# Association of Gut Microbiome Shifts With Metabolic Alterations in Prediabetes: A Cross-Sectional Study

**DOI:** 10.7759/cureus.111964

**Published:** 2026-07-02

**Authors:** Wei-Lin Chang, Cheng-Yu Chen, Jing-Hui Wu, Yi Cheng Hou

**Affiliations:** 1 Department of Nutrition, Taipei Tzu Chi Hospital, Buddhist Tzu-Chi Medical Foundation, New Taipei City, TWN; 2 Department of Family Medicine, Taipei Tzu Chi Hospital, Buddhist Tzu-Chi Medical Foundation, New Taipei City, TWN

**Keywords:** dysbiosis, gut microbiota, insulin resistance, metabolic health, prediabetes, type 2 diabetes mellitus

## Abstract

Background

Early identification and intervention are essential to prevent the progression of prediabetes to type 2 diabetes mellitus (T2DM). The gut microbiota plays a key role in host metabolism, and its dysbiosis may contribute to metabolic disorders. This study aimed to compare gut microbiota profiles between individuals with prediabetes and healthy adults and to explore their potential metabolic associations.

Materials and methods* *

A total of 117 adults aged 18-65 years were recruited, including 57 patients with prediabetes and 60 healthy controls. Demographic data and stool samples were collected. Gut microbiota composition was analyzed using 16S rRNA gene sequencing targeting the V3-V4 region. To minimize batch effects, raw sequencing data from both cohorts were processed using a unified bioinformatics pipeline.

Results* *

Individuals with prediabetes exhibited significantly lower gut microbial diversity (Simpson index, p < 0.001) and distinct microbial composition (Permutational Multivariate Analysis of Variance (PERMANOVA), p = 0.001) compared with healthy controls. Additionally, several bacterial genera differed significantly between groups, with 11 genera enriched and four genera depleted in the prediabetes group, indicating a shift in gut microbiota structure associated with prediabetes.

Conclusion* *

The gut microbiota of individuals with prediabetes differed significantly from that of healthy adults, showing reduced diversity and altered bacterial composition. These findings indicate that gut microbiota dysbiosis is associated with prediabetes-related metabolic alterations, although causal relationships cannot be inferred due to the cross-sectional design.

## Introduction

Diabetes mellitus is a major global health concern, affecting over 500 million adults worldwide [1]. Prediabetes represents an intermediate and potentially reversible stage; however, a substantial proportion of individuals progress to type 2 diabetes mellitus (T2DM) [2]. Early identification of high-risk individuals remains a clinical priority. Emerging evidence suggests that the gut microbiota plays an important role in metabolic regulation [3], yet its involvement in the early stages of dysglycemia remains incompletely understood.

The gut microbiota has been associated with glucose metabolism, inflammation, and intestinal barrier function [3,4]. Alterations in microbial composition may influence insulin resistance through mechanisms such as increased intestinal permeability and systemic inflammation [5,6]. In addition, microbial metabolites, including short-chain fatty acids (SCFAs), contribute to host metabolic homeostasis [6,7]. Previous studies have demonstrated differences in gut microbiota between patients with T2DM and healthy individuals [8]; however, findings in prediabetes remain inconsistent.

Despite growing interest in the gut microbiome, studies focusing on individuals with prediabetes, particularly in Asian populations, remain limited [8-10]. Furthermore, important confounding factors such as age, BMI, and lifestyle variables may influence gut microbiota composition, yet they are not consistently addressed in existing studies [11,12]. Therefore, further investigation is needed to better characterize microbiota alterations in prediabetes.

Accordingly, the objectives of this study were to: (1) compare gut microbiota diversity and composition between individuals with prediabetes and healthy adults in Taiwan, and (2) explore associations between microbial alterations and predicted metabolic functions inferred via Tax4Fun analysis. Given the cross-sectional design, these exploratory analyses are intended to be hypothesis-generating and to describe microbial associations rather than represent direct measurements of metabolic activity or establish causal relationships.

## Materials and methods

Study design and participants

This cross-sectional study included 60 healthy adults and 57 individuals with prediabetes. Participants with prediabetes were recruited from the Department of Family Medicine, Division of Metabolism, and Center for Preventive Medicine at Taipei Tzu Chi Hospital. Prediabetes was defined according to the American Diabetes Association (ADA) criteria as a fasting plasma glucose level of 100-125 mg/dL and a glycated hemoglobin (HbA1c) level of 5.7%-6.4% [13].

Eligible participants met the following inclusion criteria: (1) age 18-65 years and (2) no antibiotic or probiotic use within the eight weeks preceding fecal sample collection. Demographic data, including sex, age, height, weight, and BMI, were collected, and a single fecal sample was obtained from each participant. Written informed consent was obtained prior to enrollment.

Gut microbiota data from 60 healthy adults were obtained from a publicly available Taiwanese microbiome database [14]. Strict inclusion and exclusion criteria were applied to minimize potential confounding factors, including the absence of major metabolic, gastrointestinal, autoimmune, or systemic diseases. This study was approved by the Institutional Review Board of Taipei Tzu Chi Hospital, Buddhist Tzu Chi Medical Foundation (Approval Nos. 07-XD-061 and 10-XD-071).

Biochemical measurements

Fasting plasma glucose levels were measured using a Beckman Coulter AU5800 automated chemistry analyzer, and HbA1c levels were determined by high-performance liquid chromatography using the ARKRAY ADAMS A1c HA-8180V system. All measurements were conducted at the central laboratory of Taipei Tzu Chi Hospital using standardized and validated clinical laboratory procedures.

Microbiota analysis

Following fecal sample collection, specimens were stored at 4°C and centrifuged at 13,000 × g for 1 minute. The supernatant was removed, and the samples were stored at -20°C until DNA extraction. Microbial DNA was extracted using a physical bead-beating method to ensure efficient bacterial cell lysis, followed by purification with the QIAamp PowerFecal DNA Kit (QIAGEN, USA) according to the manufacturer’s instructions. The extracted DNA was quantified, and 12.5 ng of purified DNA was used for PCR amplification targeting the V3-V4 region of the 16S ribosomal RNA gene.

The PCR primers used were as follows:

Forward: 5′-TCGTCGGCAGCGTCAGATGTGTATAAGAGACAGCCTACGGGNGGCWGCAG

Reverse: 5′-GTCTCGTGGGCTCGGAGATGTGTATAAGAGACAGGACTACHVGGGTATCTAATCC

PCR products were purified using AMPure XP beads (Beckman Coulter, USA), followed by a second PCR using Nextera XT Index primers (Illumina, USA) for library preparation. The final library size was approximately 630 bp. After a second purification step, next-generation sequencing was performed using the Illumina MiSeq platform with the MiSeq Reagent Kit v3 for paired-end sequencing (2 × 250 bp). The sequencing data will be publicly available in the National Center for Biotechnology Information (NCBI) database (BioProject ID: PRJNA891488) after October 16, 2028.

Data analysis

To minimize potential batch effects arising from different sequencing sources, raw sequencing reads from both the recruited cohort and the public database were processed simultaneously using an identical bioinformatics pipeline.

Raw sequencing reads underwent quality control using the FASTX-Toolkit. The sequence quality criteria were as follows: (1) the minimum acceptable Phred quality score was 20, with more than 70% of sequence bases having a score ≥20; (2) after quality trimming from the sequence tail, sequences longer than 100 bp were retained, provided they also had an acceptable Phred quality score of 20; and (3) both forward (R1) and reverse (R2) sequencing reads that met the first and second requirements were retained for subsequent analysis. Denoising and generation of zero-radius operational taxonomic units (zOTUs) were performed using the USEARCH UNOISE algorithm [15]. Taxonomic assignment was conducted using the SILVA database (version 138) [16], with BLAST used for sequence alignment at 97% similarity.

No multivariate adjustment was performed in this exploratory analysis.

Diversity and statistical analysis

The relative abundances of taxonomically assigned zOTUs were used for downstream analyses. Alpha diversity was assessed using Simpson’s Dominance Index (D). Beta diversity was visualized using non-metric multidimensional scaling (NMDS). Differences in microbial community composition between groups were evaluated using permutational multivariate analysis of variance (PERMANOVA) [17].

Differential abundance and functional profiling

Differentially abundant taxa were identified using linear discriminant analysis effect size (LEfSe) [18]. For group comparisons, the Mann-Whitney U test or Chi-square test was applied where appropriate. To control for multiple comparisons, false discovery rate (FDR) correction was performed using the Benjamini-Hochberg procedure.

Functional metabolic pathways were predicted using Tax4Fun [19]. Statistical significance for pathway analysis was determined using ANOVA. Data visualization and statistical analyses were conducted using R (vegan, ggbiplot, and dplyr), SPSS Statistics (Version 26), and GraphPad Prism (Version 9.5). Continuous variables are expressed as median (IQR), and categorical variables as frequencies (percentages).

## Results

Participant characteristics

The descriptive characteristics of the study participants are summarized in Table 1. A total of 60 healthy adults and 57 individuals with prediabetes were included in the analysis. Importantly, no missing values were identified for any of the demographic, anthropometric, or clinical variables included in these analyses, resulting in a complete-case dataset. Participants in the prediabetes group had a significantly higher median age (healthy: 52.6 (IQR) years; prediabetes: 58.2 (IQR) years; p = 0.007) and BMI (healthy: 22.9 (IQR) kg/m²; prediabetes: 24.7 (IQR) kg/m²; p = 0.002) compared with the healthy group. No significant difference was observed in sex distribution between the two groups (healthy: 58.3% female; prediabetes: 54.4% female; p = 0.712). The observed differences in age and BMI between the two groups may act as important confounding factors and should be carefully considered, as both variables are known to influence gut microbiota composition. Therefore, these differences should be taken into consideration when interpreting the microbiota-related findings of this study.

**Table 1:** Descriptive characteristics of the study participants.

Variable	Healthy individuals (n = 60)	Patients with prediabetes (n = 57)	p-value
Age (years)	52.6 ± 14.1	58.2 ± 6.0	0.007
Sex (n (%))			
Male	25 (41.7%)	26 (45.6%)	0.712
Female	35 (58.3%)	31 (54.4%)	
BMI (kg/m²)	22.9 ± 2.2	24.7 ± 3.8	0.002
BMI category (n (%))			
Healthy weight (BMI, 18.5-24 kg/m²)	37 (61.7%)	26 (45.6%)	0.136
Overweight (BMI, 24-27 kg/m²)	23 (38.3%)	30 (52.6%)	

Alpha diversity

**Figure 1:**
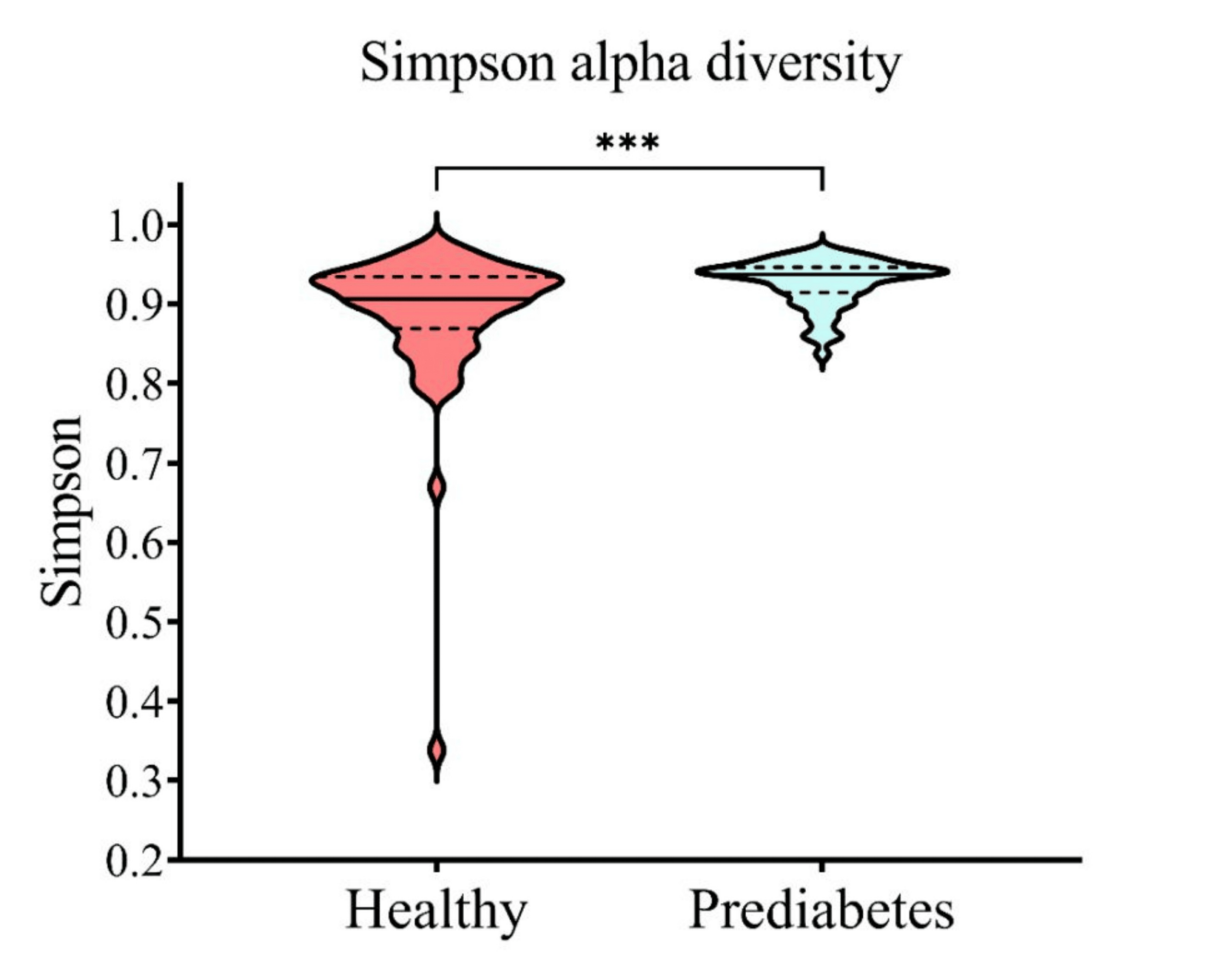
Simpson’s dominance index in healthy adults and patients with prediabetes. *p < 0.05; **p < 0.01; ***p < 0.001.

Violin plots of Simpson’s dominance index (D) for both groups are shown in Figure 1. In this index, values closer to 1 indicate greater dominance of specific taxa and lower overall microbial diversity.

The prediabetes group exhibited a significantly higher median Simpson index (0.938 (IQR)) compared with the healthy group (0.907 (IQR), p < 0.001), indicating reduced gut microbial diversity in individuals with prediabetes.

Beta diversity

**Figure 2:**
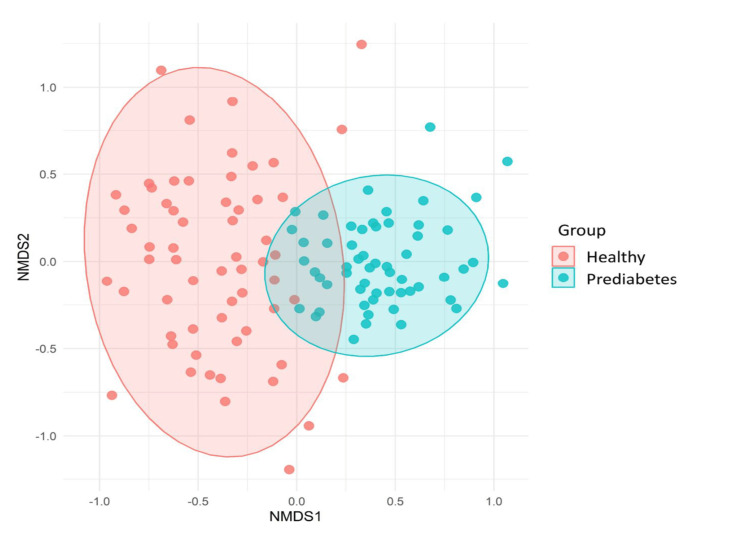
Non-metric multidimensional scaling based on Bray-Curtis dissimilarity in healthy adults and patients with prediabetes. NMDS: Non-metric multidimensional scaling.

Figure 2 shows NMDS plots illustrating beta diversity between healthy adults and individuals with prediabetes. Samples located closer together in the plot indicate greater similarity in microbial composition.

PERMANOVA revealed a significant difference in overall gut microbiota composition between the two groups (p = 0.001), indicating a distinct microbial community structure associated with prediabetes.

Taxonomic differences

Table 2 summarizes the log₂ fold changes in bacterial genera, calculated as the ratio of relative abundance in the prediabetes group to that in the healthy group. Positive values indicate higher relative abundance in the prediabetes group, whereas negative values indicate lower abundance.

**Table 2:** Changes in relative abundance at the genus level. Relative abundances were compared using the Wilcoxon rank-sum test. *Only bacterial genera exhibiting log₂ fold changes >2 or <-2 are shown.

Bacterial taxon	Log₂ fold change (Prediabetes/Healthy)	p-value
Alistipes	-2.548	<0.001
Parabacteroides	-2.52	<0.001
Phascolarctobacterium	-2.422	<0.001
Bacteroides	-2.167	<0.001
Mediterraneibacter	2.089	<0.001
Dorea	2.202	<0.001
Lachnospiraceae ND3007 group	2.205	<0.001
Clostridium	2.448	<0.001
Anaerostipes	2.521	<0.001
Anaerobutyricum hallii	2.781	<0.001
Collinsella	2.856	<0.001
Streptococcus	2.864	<0.001
Bifidobacterium	2.935	<0.001
Fusicatenibacter	3.241	<0.001
Blautia	3.871	<0.001

A total of 15 genera showed absolute log₂ fold changes greater than 2. Among these, 11 genera, *Mediterraneibacter*, *Dorea*, ND3007 group of Lachnospiraceae, *Clostridium*, *Anaerostipes*, *Eubacterium hallii* group, *Collinsella*, *Streptococcus*, *Bifidobacterium*, *Fusicatenibacter*, and *Blautia*, were relatively enriched in the prediabetes group. In contrast, four genera, *Alistipes*, *Parabacteroides*, *Phascolarctobacterium*, and *Bacteroides*, were relatively depleted.

LEfSe analysis (Table 3) further identified taxa that were differentially abundant between groups. *Bacteroides* was significantly enriched in healthy adults, whereas multiple genera, including *Blautia*, *Faecalibacterium*, *Bifidobacterium*, *Collinsella*, *Fusicatenibacter*, *Streptococcus*, *Clostridium*, *Lachnoclostridium*, *Anaerostipes*, *Mediterraneibacter*, *Butyricicoccus*, *Dorea*, *Clostridioides*, and *Eggerthella*, were significantly enriched in the prediabetes group.

**Table 3:** LEfSe analysis comparing the healthy group and the prediabetes group. LEfSe: Linear discriminant analysis effect size; LDA: Linear discriminant analysis.

Bacterial taxon	Log10	Group	LDA score (>4)	p-value
k__Bacteria;p__Bacteroidetes;c__Bacteroidia;o__Bacteroidales;f__Bacteroidaceae;g__Bacteroides	5.464	Healthy	5.024	4.56 × 10^-6^
k__Bacteria;p__Firmicutes;c__Clostridia;o__Clostridiales;f__Veillonellaceae;g__Megasphaera	4.462	Healthy	4.083	9.18 × 10^-5^
k__Bacteria;p__Firmicutes;c__Clostridia;o__Clostridiales;f__Veillonellaceae;g__Phascolarctobacterium	4.611	Healthy	4.3	2.52 × 10^-4^
k__Bacteria;p__Bacteroidetes;c__Bacteroidia;o__Bacteroidales;f__Prevotellaceae;g__Prevotella	4.788	Healthy	4.213	3.24 × 10^-2^
k__Bacteria;p__Firmicutes;c__Clostridia;o__Clostridiales;f__Lachnospiraceae;g__Blautia	4.994	Prediabetes	4.576	4.12 × 10^-11^
k__Bacteria;p__Firmicutes;c__Clostridia;o__Clostridiales;f__Lachnospiraceae;g__Coprococcus	4.629	Prediabetes	4.102	6.64 × 10^-6^
k__Bacteria;p__Firmicutes;c__Clostridia;o__Clostridiales;f__Ruminococcaceae;g__Faecalibacterium	4.899	Prediabetes	4.426	3.24 × 10^-5^
k__Bacteria;p__Actinobacteria;c__Actinobacteria;o__Bifidobacteriales;f__Bifidobacteriaceae;g__Bifidobacterium	4.816	Prediabetes	4.295	1.69 × 10^-4^
k__Bacteria;p__Firmicutes;c__Bacilli;o__Lactobacillales;f__Streptococcaceae;g__Streptococcus	4.5	Prediabetes	4.091	4.61 × 10^-4^
k__Bacteria;p__Actinobacteria;c__Coriobacteriia;o__Coriobacteriales;f__Coriobacteriaceae;g__Collinsella	4.514	Prediabetes	4.041	5.04 × 10^-3^

Overall, these findings indicate compositional differences in gut microbiota between individuals with prediabetes and healthy controls.

Functional pathway analysis

Table 4 presents the predicted functional profiles of the gut microbiota based on Tax4Fun analysis.

**Table 4:** Functional pathway analysis comparing the healthy group and the prediabetes group. KEGG: Kyoto Encyclopedia of Genes and Genomes.

Comparison	KEGG ID	KEGG pathway name	Fold change (Healthy/Prediabetes)	p-value
Healthy/Prediabetes	ko04975	Fat digestion and absorption	6.361	6.31 × 10^-5^
	ko00062	Fatty acid elongation	0.172	4.22 × 10^-14^
	ko00072	Synthesis and degradation of ketone bodies	0.453	7.30 × 10^-12^
	ko00563	Glycosylphosphatidylinositol (GPI)-anchor biosynthesis	64.972	1.52 × 10^-2^
	ko00604	Glycosphingolipid biosynthesis - ganglio series	2.04	9.55 × 10^-10^

In lipid metabolism-related pathways, the healthy group showed higher predicted functional potential in pathways such as “fat digestion and absorption,” “fatty acid elongation,” and “ketone body synthesis and degradation” compared with the prediabetes group.

Similarly, for glucose metabolism-related pathways, higher predicted functional potential in pathways including “glycosylphosphatidylinositol-anchor biosynthesis” and “glycosphingolipid biosynthesis-ganglio series” was observed in healthy individuals.

These findings suggest potential differences in microbiota-associated metabolic functions between groups; however, they are based on computational predictions and should be interpreted with caution, as they do not represent direct measurements of microbial metabolic activity.

## Discussion

Gut microbiota dysbiosis has been widely reported to be associated with T2DM, potentially through mechanisms involving altered insulin regulation and chronic low-grade inflammation [20,21].

In the present study, gut microbiota diversity and composition differed significantly between individuals with prediabetes and healthy adults, consistent with previous findings in T2DM populations [22]. Prior studies have suggested that modulation of the gut microbiota may influence host metabolic function. For instance, fecal microbiota transplantation from healthy donors to individuals with metabolic syndrome has been shown to improve insulin sensitivity [23]. In addition, the gut microbiota has been reported to interact with host metabolism through pathways involving SCFAs, bile acids, and incretin hormones such as glucagon-like peptide-1 [24].

The reduced microbial diversity observed in the prediabetes group is consistent with previous reports linking lower diversity to insulin resistance and metabolic disorders [25]. Notably, participants with prediabetes also had a significantly higher BMI, which may partially contribute to the observed differences in gut microbiota composition. Therefore, these findings should be interpreted with consideration of potential confounding factors.

Differential abundance and functional analysis

LEfSe analysis identified multiple genera that were differentially abundant between groups. The enrichment of *Bacteroides* in healthy individuals was observed in the present study. In contrast, several genera, including *Blautia*, *Faecalibacterium*, *Bifidobacterium*, *Collinsella*, *Fusicatenibacter*, *Streptococcus*, *Clostridium*, *Lachnoclostridium*, *Anaerostipes*, *Mediterraneibacter*, *Butyricicoccus*, *Dorea*, *Clostridioides*, and *Eggerthella*, were enriched in the prediabetes group, consistent with prior reports linking these taxa to metabolic disorders [3,9,10]. However, the functional roles of these genera may be context-dependent and vary across populations; therefore, they should be interpreted with caution.

Functional pathway analysis using Tax4Fun suggested differences between the healthy and prediabetes groups in predicted functional potential in pathways related to lipid and glucose metabolism [26-28]. However, it is important to emphasize that these findings are based on computational predictions derived from 16S rRNA data and do not represent direct measurements of microbial metabolic activity. Therefore, these results should be interpreted cautiously.

Detailed analysis of genus-level changes

This study confirmed a significant divergence in gut microbiota composition between healthy individuals and individuals with prediabetes, consistent with findings from both intra- and inter-ethnic studies [11,12]. Genus-level analysis further identified several taxa enriched in the prediabetes group, including *Mediterraneibacter*, *Dorea*, Lachnospiraceae ND3007 group, *Clostridium*, *Anaerostipes*, *Eubacterium hallii *group, *Collinsella*, *Streptococcus*, *Bifidobacterium*, *Fusicatenibacter*, and *Blautia*.

Some of these genera have been previously associated with metabolic dysfunction. For example, *Collinsella* has been linked to obesity, nonalcoholic fatty liver disease, and impaired glucose metabolism [29,30], whereas *Dorea* and *Blautia* have been associated with inflammatory processes [31,32]. In addition, *Mediterraneibacter* has been reported to exhibit functional heterogeneity across strains, including roles related to SCFA production [33]. However, these associations are not entirely consistent across studies, and their functional roles remain incompletely understood.

Several enriched genera, such as Lachnospiraceae ND3007 group, *Anaerostipes*, and *Eubacterium hallii* group, are commonly associated with metabolic regulation and SCFA production [34-36]. Although SCFAs are considered beneficial for metabolic health, the relationships between these taxa and metabolic outcomes appear to be complex and context-dependent. In addition, the observed enrichment of *Clostridium* in the prediabetes group differs from some previous reports suggesting beneficial metabolic effects of certain *Clostridium* species [37], highlighting variability across studies.

Notably, the increased abundance of *Bifidobacterium* in the prediabetes group contrasts with its generally reported beneficial metabolic effects [38]. This discrepancy may reflect population-specific factors, dietary influences, or functional heterogeneity at the species level and warrants further investigation.

Conversely, several genera, including *Alistipes*, *Parabacteroides*, *Phascolarctobacterium*, and *Bacteroides*, were depleted in the prediabetes group. These taxa are known to be associated with metabolic regulation and host metabolic homeostasis [39-42]. Their reduced abundance may be associated with impaired metabolic regulation; however, causality cannot be inferred from this cross-sectional study.

Overall, although some findings are consistent with previous reports, discrepancies remain across studies. These differences may reflect variations in study populations, dietary patterns, and analytical methods. Therefore, further longitudinal and mechanistic studies are required to clarify the roles of specific microbial taxa in the development of prediabetes.

Causality and intervention

Current evidence suggests a bidirectional relationship between gut microbiota dysbiosis and metabolic disorders, although the temporal sequence remains unclear. It is not yet established whether alterations in gut microbiota precede the development of prediabetes or occur as a consequence of metabolic dysfunction.

In individuals with prediabetes, gut microbiota composition may shift from a normobiotic state, potentially in association with metabolic changes. These alterations may be influenced by modifiable factors such as diet and lifestyle. Accordingly, lifestyle interventions, including dietary modification, may contribute to improvements in gut microbiota composition and could potentially delay the progression to diabetes. However, these relationships remain to be validated in longitudinal and interventional studies.

Diet is considered an important determinant of gut microbiota composition, as demonstrated in both animal and human studies [43]. Previous research suggests that dietary factors account for a substantial proportion of microbiota variability, whereas host genetics contributes to a lesser extent. Dietary fiber, which serves as a primary substrate for gut microbiota, is classified as a prebiotic [44]. Its fermentation by gut bacteria leads to the production of SCFAs, which have been associated with intestinal barrier function and host metabolic regulation.

In addition to SCFAs, the gut microbiota has been reported to interact with host metabolism through multiple pathways, including gut peptide signaling and inflammatory processes. However, these mechanisms were not directly assessed in the present study and should be interpreted as literature-based associations rather than causal relationships.

Limitations

Despite the insights provided, several limitations should be acknowledged.

First, the use of a public database for the healthy control cohort may introduce heterogeneity in sample collection, DNA extraction, and sequencing procedures compared with those used for the recruited participants. These methodological differences may contribute to potential batch effects. To mitigate this issue, raw sequencing data from both cohorts were reprocessed using a unified bioinformatics pipeline. However, residual technical variability cannot be entirely excluded.

Second, although the healthy participants underwent rigorous screening, detailed biochemical data, including fasting plasma glucose and HbA1c levels, were not available for this group. Nevertheless, the strict exclusion criteria, such as the absence of obesity (BMI >27 kg/m²), metabolic disorders, and recent medication use, likely reduced the probability of including individuals with undiagnosed prediabetes.

Third, this study employed a cross-sectional design, which limits the ability to infer causal relationships between gut microbiota alterations and the progression of prediabetes. In addition, potential confounding factors, including differences in age and BMI, lifestyle behaviors (e.g., smoking and physical activity), and probiotic use, were not fully controlled and may have influenced the observed microbial patterns. Although recent probiotic use was restricted by the inclusion criteria, detailed information regarding long-term consumption of probiotic-containing products was not systematically collected, which may represent a source of residual confounding. Furthermore, multivariable analyses adjusting for potential confounders such as age and BMI were not performed in this exploratory study and should be considered in future research. Because of this lack of multivariable adjustment, we cannot exclude the possibility that some of the observed microbial differences reflect adiposity-related pathophysiological pathways or lifestyle variations rather than prediabetes status alone. Future well-matched, longitudinal cohorts utilizing multivariable regression models are required to disentangle these intertwined metabolic influences and validate our findings.

Finally, dietary intake data were not comprehensively assessed, despite diet being a major determinant of gut microbiota composition. Additionally, the relatively modest sample size may limit the generalizability of the findings. Future studies should incorporate detailed dietary assessments, larger and well-matched cohorts, longitudinal designs, and multivariable regression analyses to better control for potential confounding variables such as age and BMI, thereby further validating these findings and clarifying the role of gut microbiota in metabolic disease progression.

## Conclusions

This study suggests that the gut microbial composition of individuals with prediabetes differs from that of healthy adults, with distinct taxonomic shifts observed between groups. Individuals with prediabetes exhibited reduced gut microbiota diversity compared with healthy controls.

Genus-level analysis identified 15 taxa with an absolute log₂ fold change >2.0, including 11 genera enriched in the prediabetes group (*Mediterraneibacter*, *Dorea*, Lachnospiraceae ND3007 group, *Clostridium*, *Anaerostipes*, *Eubacterium hallii* group, *Collinsella*, *Streptococcus*, *Bifidobacterium*, *Fusicatenibacter*, and *Blautia*) and four genera that were relatively depleted (*Alistipes*, *Parabacteroides*, *Phascolarctobacterium*, and *Bacteroides*).

These findings indicate an association between gut microbiota alterations and prediabetes. However, further longitudinal and mechanistic studies are required to clarify the causal relationships and potential clinical implications of these microbial changes.
